# The Effects of Temperature and Relative Humidity on the Viability of the SARS Coronavirus

**DOI:** 10.1155/2011/734690

**Published:** 2011-10-01

**Authors:** K. H. Chan, J. S. Malik Peiris, S. Y. Lam, L. L. M. Poon, K. Y. Yuen, W. H. Seto

**Affiliations:** Department of Microbiology, The University of Hong Kong, Queen Mary Hospital, Pokfulam, Hong Kong

## Abstract

The main route of transmission of SARS CoV infection is presumed to be respiratory droplets. However the virus is also detectable in other body fluids and excreta. The stability of the virus at different temperatures and relative humidity on smooth surfaces were studied. The dried virus on smooth surfaces retained its viability for over 5 days at temperatures of 22–25°C and relative humidity of 40–50%, that is, typical air-conditioned environments. However, virus viability was rapidly lost (>3 log_10_) at higher temperatures and higher relative humidity (e.g., 38°C, and relative humidity of >95%). The better stability of SARS coronavirus at low temperature and low humidity environment may facilitate its transmission in community in subtropical area (such as Hong Kong) during the spring and in air-conditioned environments. It may also explain why some Asian countries in tropical area (such as Malaysia, Indonesia or Thailand) with high temperature and high relative humidity environment did not have major community outbreaks of SARS.

## 1. Introduction

Severe acute respiratory syndrome (SARS), was a new emerging disease associated with severe pneumonia and spread to involve over 30 countries in 5 continents in 2003. A novel coronavirus was identified as its cause [1–3]. SARS had a dramatic impact on health care services and economies of affected countries, and the overall mortality rate was estimated to be 9%, but rising to 50% in those aged 60 or above [4]. A notable feature of this disease was its predilection for transmission in the health care setting and to close family and social contacts. The disease is presumed to be spread by droplets, close direct or indirect contact, but the relative importance of these routes of transmission is presently unclear. A study showed that viral aerosol generation by a patient with SARS was possible and therefore airborne droplet transmission was a possible means of transmission [5]. However, the role of fomites and environmental contamination in transmission of infection is presently still unclear. An outbreak of disease affecting over 300 residents in high-rise apartment block (Amoy Gardens) in Hong Kong could not be explained by respiratory droplet transmission from infected patients [6]. Infectious virus is detectable in the faeces [7], and aerosolization of virus in contaminated faeces is believed to be the mode of transmission of this outbreak [8]. 

We and others have reported that infectivity of SARS CoV (SARS coronavirus) was lost after heating at 56°C for 15 minutes but that it was stable for at least 2 days following drying on plastic. It was completely inactivated by common fixatives used in laboratory [9, 10]. Another study showed that it was inactivated by ultraviolet light, alkaline (pH > 12), or acidic (pH < 3) conditions [11]. Human coronaviruses have been shown to survive in PBS or culture medium with 5–10% FCS for several days [12–14] but they only survive a few hours after drying [13, 14]. There have been some studies reporting an association between the SARS outbreak, metrological factors, and air pollution [15–17]. Thus, information on the survival of the SARS coronavirus (SCoV) in the environment at different temperature and humidity conditions is of significant interest to understanding virus transmission. A recent study using surrogate coronaviruses (transmissible gastroenteritis virus (TGEV) and mouse hepatitis virus (MHC)) has investigated the effect of air temperature and relative humidity on coronavirus survival on surface [18]. The survival effects of these environmental factors on SARS coronavirus remain unclear. In the present study, we report the stability of the SARS coronavirus at different temperatures and relative humidity.

## 2. Material and Methods

### 2.1. Virus Strain and Cell Line

The SARS CoV strain used in this study is HKU39849. Foetal monkey kidney cells (FRhK-4) were cultured in minimal essential medium (MEM, Gibco, USA) with 10% foetal calf serum and penicillin streptomycin (Gibco, USA) at 37°C in 5% CO_2_ and were used for growing stock virus and for titration of viral infectivity [1, 2]. 

### 2.2. Preparation of Stock Virus

Stock virus was harvested when infection approximately 75% of the cell monolayer of a virus infected flask manifested cytopathic effect (CPE). Infected cells were subjected to one cycle of freeze and thaw centrifuged at 2000 rpm for 20 minutes to remove cell debris and the culture supernatant was aliquoted and stored at −80°C until use.

### 2.3. Determination of Tissue Culture Infectious Dose (50%) (TCID_50_)

96-well microtitre plates containing 100 *μ*L of confluent FRhK-4 were infected with 100 *μ*L of serial 10-fold of dilutions of stock virus in minimal essential medium with 1% FCS (maintenance medium) starting from 10^−1^ to 10^8^. Titrations were done in quadruplicate. Infected cells were incubated for 4 days at 37°C. Appearance of CPE was recorded daily. TCID_50_ was determined according to Reed and the Muench method [19]. 

### 2.4. Effect of Drying, Heat, and Relative Humidity

Ten microlitre of maintenance medium containing 10^7^ TCID_50_ per mL of virus was placed in individual wells of a 24-well plastic plates and allowed to dry at room temperature (22~25°C) and relative humidity of 40–50% (i.e., conditions prevailing in a typical air-conditioned room). One hundred microlitre of MM was used to resuspend the virus at 0 hr, 3 hr, 7 hr, 11 hr, 13 hr, 24 hr, and up to 4 weeks and the residual virus infectivity was titrated. Controls in closed screw cap eppendorf tube were included each time and treated similarly but without drying.

The experiment was repeated at different temperatures (38°C, 33°C, 28°C) and relative humidities (>95%, 80~89%) for 3 hr, 7 hr, 11 hr, 13 hr, and 24 hr. A nebulizer under a controlled condition was used to generate high and relative low humidity environment. All the experiments above were conducted in duplicate and the residual viral infectivity was titrated. 

### 2.5. Infectivity Assay

The infectivity of residual virus was titrated in quadruplicate on 96-well microtitre plates containing 100 *μ*L of confluent FRhK-4 cells. 100 *μ*L of serial 10-fold of dilutions of virus in maintenance medium starting from 10^−1^ to 10^8^ was added into FRhK-4 cells. The infected cells were incubated at 37°C for 4 days. Appearance of CPE was recorded daily. TCID_50_ was determined according to the Reed and Muench method [19]. 

## 3. Results

**Figure 1:**
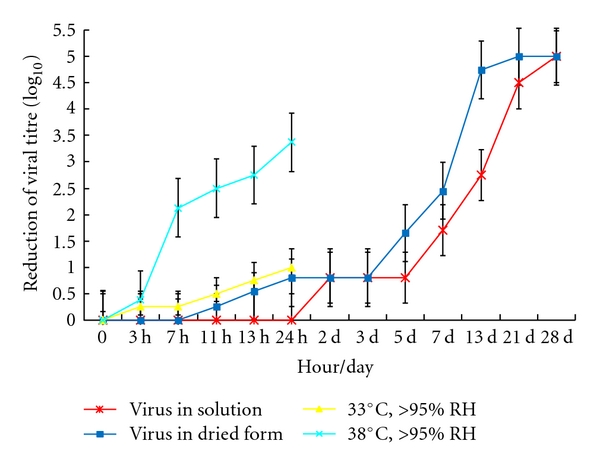
Residual virus infectivity at 22–25°C with relative humidity 40–50% (starting titre 10^5^/10 *μ*L) and at 33°C or 38°C with relative humidity >95%.

Ten microlitre of 10^7^ TCID_50_ per mL of virus was placed in individual wells of a 24-well plastic plate (representing a nonporous surface) and dried. The dried virus was then incubated at different temperatures (38°C, 33°C, 28°C) at different relative humidity (>95%, 80~89%) for 3 hr, 7 hr, 11 hr, 13 hr, and 24 hr and the residual viral infectivity was titrated. A similar experiment was conducted at room temperature and relative humidity of about 40–50% (air-conditioned room) for up to 4 weeks. Virus dried on plastic retained viability for up to 5 days at 22~25°C at relative humidity of 40~50% with only 1 log⁡_10_ loss of titre (Figure 1). After that virus infectivity is gradually lost ever time. Loss of virus infectivity in solution was generally similar to dried virus under these environmental conditions. This indicates that SARS CoV is a stable virus that may potentially be transmitted by indirect contact or fomites, especially in air-conditioned environments.  

**Figure 2:**
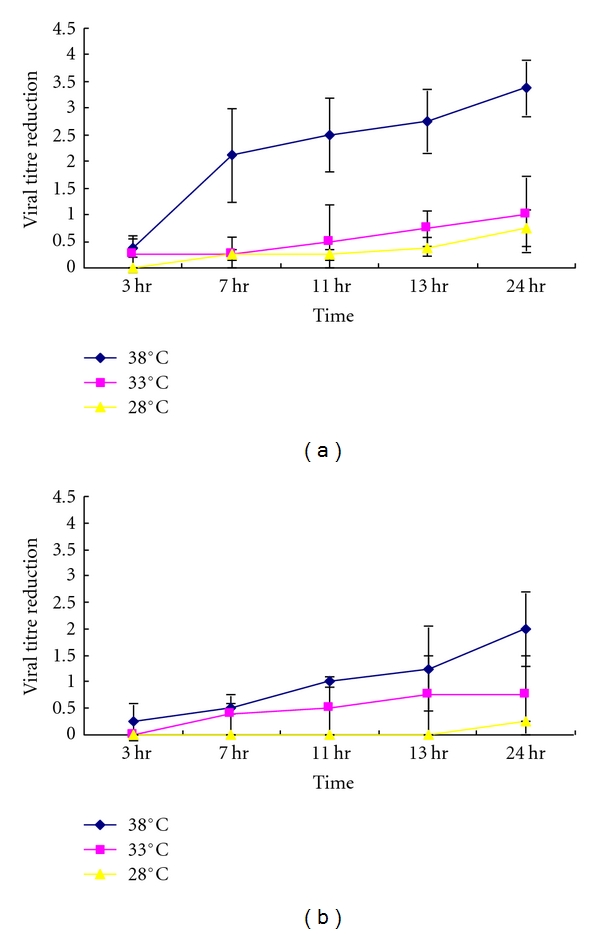
Infectivity of SARS Coronavirus (10^5^/10 *μ*L) to different temperatures at (a) >95% relative humidity, (b) >80–89%.

**Figure 3:**
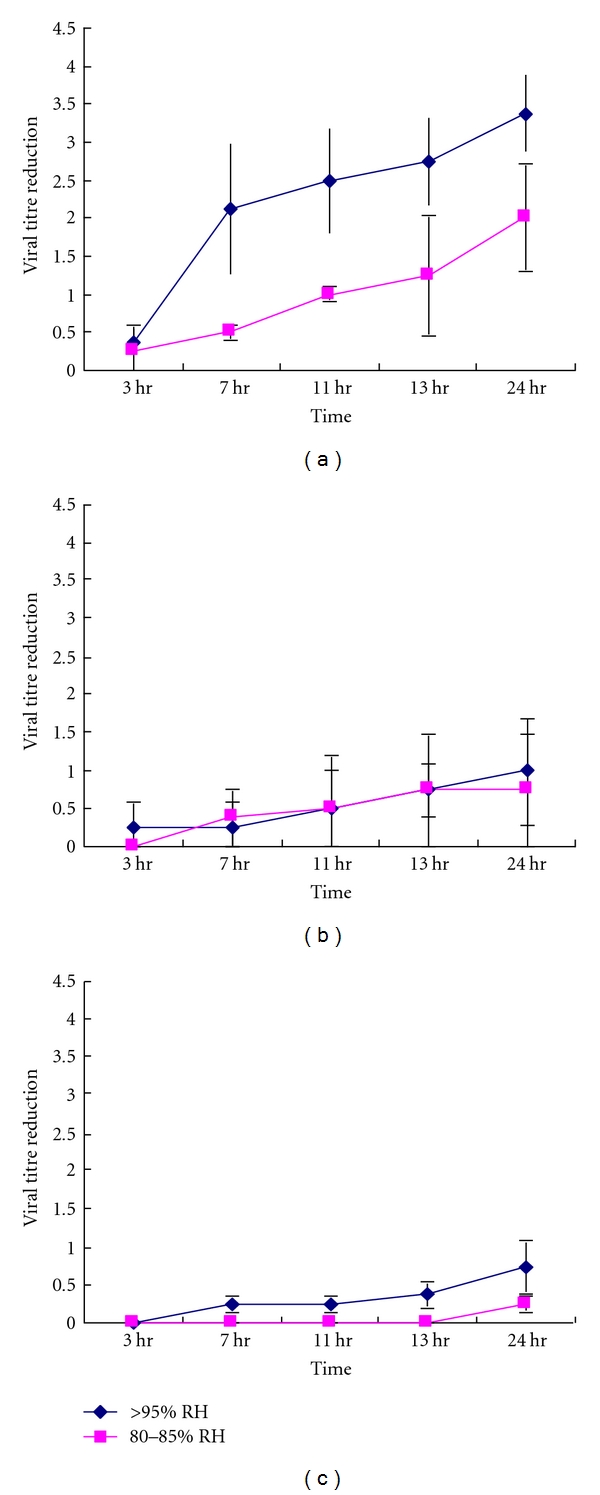
Infectivity of SARS Coronavirus (starting titre 10^5^/10 *μ*L) at different relative humidity at (a) 38°C, (b) 33°C, and (c) 28°C.

High relative humidity (>95%) at comparatively low temperature (28°C and 33°C) did not affect the virus infectivity significantly (Figure 2(a)). High temperature (38°C) at 80–90% relative humidity led to a 0.25~2  log⁡_10_ loss of titre at 24 hr (Figure 2(b)). However, if the dried virus was stored at high temperature (38°C) and high relative humidity (>95%), there was a further ~1.5 log⁡ loss of titre for each time point up to 24 hr (0.38~3.38 log⁡_10_) when compared with high temperature (38°C) at a lower relative humidity 80–90% (Figures 3(a)–3(c)). 

## 4. Discussion

Viruses do not replicate outside living cell but infectious virus may persist on contaminated environmental surfaces and the duration of persistence of viable virus is affected markedly by temperature and humidity. Contaminated surfaces are known to be significant vectors in the transmission of infections in the hospital setting as well as the community. The role of fomites in the transmission of RSV has been clearly demonstrated [20]. Survival of viruses on a variety of fomites has been studied for influenza viruses, paramyxoviruses, poxviruses, and retroviruses [21]. The human coronavirus associated with the common cold was reported to remain viable only for 3 hours on environmental surfaces after drying, although it remains viable for many days in liquid suspension [13]. Parainfluenza and RSV viruses were viable after drying on surfaces for 2 and 6 hours, respectively [20, 22]. In aerosolised form, human coronavirus 229E is generally less stable in high humidity [12]. The environmental stability of SCoV was previously unknown and this information is clearly important for understanding the mechanisms of transmission of this virus in a hospital and community setting. 

In the present study, we have demonstrated that SARS CoV can survive at least two weeks after drying at temperature and humidity conditions found in an air-conditioned environment. The virus is stable for 3 weeks at room temperature in a liquid environment but it is easily killed by heat at 56°C for 15 minutes [9]. This indicates that SARS CoV is a stable virus that may potentially be transmitted by indirect contact or fomites. These results may indicate that contaminated surfaces may play a major role in transmission of infection in the hospital and the community. 

Our studies indicate that SCoV is relatively more stable than the human coronaviruses 229E or OC43 and some other viral respiratory pathogens such as respiratory syncytial virus. These findings suggest that, while direct droplet transmission is an important route of transmission [23], the role of fomites and environmental contamination in virus transmission may play a significant role in virus transmission. In particular, fomites may contribute to the continued transmission of infection in the nosocomial setting that continues to occur in spite of the great attention and stringent precautions taken to prevent droplet spread. In addition to droplet precautions, reenforcing contact precautions and hand washing is called for. 

Faecal contamination of SCoV coronavirus may thus be an effective route of transmission of the disease. The outbreak in Amoy Garden in Hong Kong which affected over 300 residents in a single-apartment block with thought to have been transmitted by contaminated sewage. The stability of the virus on environmental surfaces and its presence in faeces indicates the potential that fecal contamination of fresh-food production may pose a threat for virus transmission; especially in countries with poor sanitation and sewage disposal systems and that studies to address this possibility are needed. 

In this study, we showed that high temperature at high relative humidity has a synergistic effect on inactivation of SARS CoV viability while lower temperatures and low humidity support prolonged survival of virus on contaminated surfaces. The environmental conditions of countries such as Malaysia, Indonesia, and Thailand are thus not conducive to the prolonged survival of the virus. In countries such as Singapore and Hong Kong where there is a intensive use of air-conditioning, transmission largely occurred in well-air-conditioned environments such as hospitals or hotels. Further, a separate study has shown that during the epidemic, the risk of increased daily incidence of SARS was 18.18-fold higher in days with a lower air temperature than in days with a higher temperature in Hong Kong [24] and other regions [15–17]. Taken together, these observations may explain why some Asian countries in tropical area (with high temperature at high relative humidity) such as Malaysia, Indonesia, and Thailand did not have nosocomial outbreaks of SARS (Tables 1 and 2(a)–2(c)). It may also explain why Singapore, which is also in tropical area (Table 2(d)), had most of its SARS outbreaks in hospitals (air-conditioned environment). Interestingly, during the outbreak of SARS in Guangzhou, clinicians kept the windows of patient rooms open and well ventilated and these may well have reduced virus survival and this reduced nosocomial transmission. SARS CoV can retain its infectivity up to 2 weeks at low temperature and low humidity environment, which might facilitate the virus transmission in community as in Hong Kong which locates in subtropical area (Table 2(e)). Other environmental factors including wind velocity, daily sunlight, and air pressure, had shown to be associated with SARS epidemic, should also be considered [16, 17]. The dynamics of SARS epidemic involves multiple factors including physical property of virus, outdoor and indoor environments, hygiene, space, and genetic predispositions [10, 15–17, 24, 25]. Understanding the stability of viruses in different temperature and humidity conditions is important in understanding transmission of novel infectious agent including that of the recent influenza Apandemic H1N12009. 

## Figures and Tables

**Table 1:** WHO SARS report—based on data as of the 31st December 2003.

Areas	Total	Medan age	Deaths	Case fatality Ratio (%)	No. of imported Cases (%)	No. of HCW (%)	First case	Last case
China	5327	NKn	349	7	NA	1002 (19)	Nov-02	Jun-03
Hong Kong	1755	40	299	17	NA	386 (22)	Feb-03	May-03
Taiwan	346	42	37	11	21 (6)	68 (20)	Feb-03	Jun-03
Singapore	238	35	33	14	8 (3)	97 (41)	Feb-03	May-03
Viet Nam	63	43	5	8	1 (2)	36 (57)	Feb-03	Apr-03
Indonesia	2	56	0	0	2 (100)	0 (0)	Apr-03	Apr-03
Malaysia	5	30	2	40	5 (100)	0 (0)	Mar-03	Apr-03
Thailand	9	42	2	22	9 (100)	1 (11)	Mar-03	May-03
Philippines	14	41	2	14	7 (50)	4 (29)	Feb-03	May-03

Total	8096		774	9.6	142	1706 (21)		

**Table 2:** A summary of the meteorological data of 2005 in average weather conditions*.

Month	Average sunlight (hours)	Temperature		Discomfort from heat and humidity	Relative humidity	
		Min	Max		am	pm
(a) Kuala Lumpur, Malaysia						
Jan	6	22	32	High	97	60
Feb	7	22	33	High	97	60
March	7	23	33	High	97	58
April	6	23	33	High	97	63
May	6	23	33	High	97	66
June	7	22	33	High	96	63
July	7	23	32	High	95	63
Aug	6	23	32	High	96	62
Sept	6	23	32	High	96	64
Oct	5	23	32	High	96	65
Nov	5	23	32	High	97	66
Dec	5	22	32	High	97	61

(b) Jakarta, Indonesia						
Jan	5	23	29	High	95	75
Feb	5	23	29	High	95	75
March	6	23	30	High	94	73
April	7	24	31	High	94	71
May	7	24	31	High	94	69
June	7	23	31	High	93	67
July	7	23	31	High	92	64
Aug	8	23	31	High	90	61
Sept	8	23	31	High	90	62
Oct	7	23	31	High	90	64
Nov	6	23	30	High	92	68
Dec	5	23	29	High	92	71

(c) Bangkok, Thailand						
Jan	9	20	32	High	91	53
Feb	8	22	33	High	92	55
March	9	24	34	High	92	56
April	8	25	35	Extreme	90	58
May	8	25	34	Extreme	91	64
June	6	24	33	Extreme	90	67
July	5	24	32	High	91	66
Aug	5	24	32	High	92	66
Sept	5	24	32	High	94	70
Oct	6	24	31	High	93	70
Nov	8	22	31	High	92	65
Dec	9	20	31	High	91	56

(d) Singapore						
Jan	5	23	30	High	82	78
Feb	7	23	31	High	77	71
March	6	24	31	High	76	70
April	6	24	31	High	77	74
May	6	24	32	Extreme	79	73
June	6	24	31	High	79	73
July	6	24	31	High	79	72
Aug	6	24	31	High	78	72
Sept	5	24	31	High	79	72
Oct	5	23	31	High	78	72
Nov	5	23	31	High	79	75
Dec	4	23	31	High	82	78

(e) Hong Kong						
Jan	5	13	18	—	77	66
Feb	4	13	17	—	82	73
March	3	16	19	—	84	74
April	4	19	24	Medium	87	77
May	5	23	28	Medium	87	78
June	5	26	29	High	86	77
July	8	26	31	High	87	77
Aug	6	26	31	High	87	77
Sept	6	25	29	High	83	72
Oct	7	23	27	Medium	75	63
Nov	7	18	23	Moderate	73	60
Dec	6	15	20	—	74	63

*Data is available at BBC weather website (http://www.bbc.co.uk/weather/world/city_guides/results).
